# A gamified approach to burn education among Brazilian youth: The “Burn Game” pilot study

**DOI:** 10.1016/j.clinsp.2026.100899

**Published:** 2026-03-10

**Authors:** Felipe Giraldo Alvarez Gonçalves, Vinicius Oberdan Souza Reis, Vinicius Freire Costa Alves, Bruno Damico Terada, José Maria Soares Junior, Patricia Zen Tempski, Sérgio Henrique Bastos Damous, Edivaldo Massazo Utiyama, Rolf Gemperli, Cristina Pires Camargo

**Affiliations:** a Faculdade de Medicina, Universidade de São Paulo, São Paulo, SP, Brazil; b Disciplina de Ginecologia, Departamento de Obstetrícia e Ginecologia, Hospital das Clínicas, Faculdade de Medicina, Universidade de São Paulo, São Paulo, SP, Brazil; c Center for Development of Medical Education, Faculdade de Medicina da Universidade de São Paulo, São Paulo, SP, Brazil; d Hospital das Clínicas da Faculdade de Medicina da Universidade de São Paulo, Disciplina de Cirurgia Geral e Trauma, São Paulo, SP, Brasil; e Laboratory of Plastic Surgery and Regenerative Medicine, Faculdade de Medicina, Universidade de São Paulo, São Paulo, SP, Brazil

**Keywords:** Gamification, Burn, Education

## Abstract

**Introduction:**

Burn injuries are a significant global health concern, with an estimated 8.4 million cases annually. Although specific cost data from Brazil are limited, international reports estimate an average burn-related hospitalization cost of USD 88,218. Educational tools for populations are desirable to prevent burn injury. Thus, the authors designed a gamified model to promote burn education and prevention among Brazilian students.

**Methods:**

The intervention was conducted in four public schools, three High Schools (HS) and one Vocational School (VS). Students first completed a 21-question online pre-test assessing their baseline knowledge. A 20-minute lecture followed, covering burn classification and prevention. Students then participated in an interactive online trivia game (Burn Game) designed to reinforce learning. Finally, they completed the same test as a post-intervention assessment.

**Results:**

A total of 134 students participated (73 from VS, 61 from HS), with a mean age of 18.6 ± 4.9 years; 73.13% were female. Post-test scores improved significantly across the participants, with 132 students showing higher scores. The overall mean score difference was 7.16-points (95% CI: 6.47–7.86; p < 0.001). Subgroup analysis revealed gains in both VS (MD = 6.08; 95% CI: 5.33–6.83) and HS students (MD = 8.46; 95% CI: 7.41–9.51), with HS students showing the greatest improvement.

**Conclusion:**

The present study suggests that gamified teaching indeed contributed to significantly increasing students' knowledge about burn prevention and classification.

## Highlights

• Burn injuries affect approximately 8.4 million people annually, with 90% occurring in low- and middle-income countries.
• The study introduced a gamified learning model that combined a lecture and an interactive trivia game to educate Brazilian students on burn classification and prevention.
• This study showed a significant improvement in student knowledge, 98% of the students showed improved scores after the intervention.
• High school students demonstrated the highest gain of knowledge, surpassing vocational school students, confirming the effectiveness of the gamified strategy across different educational settings.

## Introduction

Burn Injuries (BIs) are the fourth most prevalent type of trauma worldwide, affecting approximately 8.4 million people annually, with 90% of cases occurring in low- and middle-income countries.^1^^,^^2^ In Brazil, BIs also represent a significant public health concern, with an estimated one million cases reported annually, according to the Brazilian Ministry of Health in 2014.^3^ Although specific data on burn treatment costs in Brazil is lacking, reports from the high-income countries indicate that the average cost of burn-related hospitalizations is USD 88,218,^4^ underscoring the financial and healthcare challenges associated with managing such injuries.

According to the American National Fire Protection Association, more than 3,000 deaths in the United States were attributed to Burn Injuries (BIs) in 2016.^5^ In contrast, the World Health Organization reported that burn-related mortality worldwide reached 180,000 cases that same year, with most fatalities occurring in low- and middle-income countries.^6^ Children are particularly vulnerable, accounting for 48% of all burn cases, primarily due to scald injuries.^7^ Moreover, 95% of burns are accidental, with 73% occurring in domestic settings and 8% in workplaces.^5^ Nearly all burn injuries are considered preventable.^8^

Given these concerning statistics, new strategies to prevent burns, particularly in low-income countries, have been emerging. Recently, gamified models have gained attention, as they are particularly appealing to Generation Z,^9^, ^10^, ^11^, ^12^ who exhibit a strong preference for digital applications and internet-based activities. While some trials have attempted to implement such approaches, they encountered high attrition rates, likely due to the impersonal nature of the gamified format.^9^

Therefore, this study aims to evaluate the effectiveness of an in-person gamified educational intervention in teaching young students about burn education, prevention and the identification of key factors for appropriately referring burn victims to healthcare services.

## Materials and methods

This study is a prospective non-randomized trial with Pre and Post-tests analyses, approved by the University of São Paulo Medical School’s IRB, under the protocol 66747223.8.0000.0068. The authors followed the Consort guideline, and all included participants provided an appropriate Informed Consent Form (ICF).

This study was developed as an interactive educational activity for students. The team prepared a lecture consisting of approximately 30 slides covering the definition of BIs, their types and classification, preventive measures, and pre-hospital care.

### Eligibility criteria

Only public schools were considered relevant for inclusion in this study. Specifically, the authors chose public schools that were easily reachable by the public transport network. Schools in diverse neighborhoods were included to avoid locally related biases. Finally, only schools offering (1) High School courses or (2) Vocational School courses were included.

Students were excluded if they: (1) Did not attend school on the day of the trial, (2) Did not provide a complete ICF, or (3) Were unable to participate in the activities for any medical or social reasons.

### Recruitment

Student recruitment was conducted entirely through the schools. The authors contacted the principals of the selected schools, presenting them with the proposed activity for their students and the ICF, clearly explaining that the data collected would later be used for scientific purposes. Following the proposal, the selected principals assigned us classes with at least 15 students.

### Burn game activity

In each school, the authors implemented a 90-minute gamified activity that included a 20-minute interval between the second and third segments. The activity was conducted in accordance with a structured pedagogical approach. The session began with students completing an online Pre-Test, a form containing 21 questions on BIs prevention, classification, and the identification of key factors that could guide the appropriate referral of burn victims to healthcare services. This process took about 20-minutes and was conducted on Google Forms (Google. (2025). Google Forms. Available on https://forms.google.com). The form served as a self-controlled group. At this stage, students did not receive their scores or feedback on correct and incorrect answers.

The activity itself was designed in two key steps to measure short-term knowledge acquisition. The first step was a concise lecture, lasting around 20-minutes, presented with slides. This lecture covered essential topics, including BIs prevention, classification, pre-hospital care, and the importance of seeking medical attention in case of BIs. The content was directly aligned with the questions from the Pre-Test, ensuring that all necessary information was addressed.

The second step introduced a gamified component to reinforce learning. Students formed groups of 6- to 9-members and created unique characters within the online trivia game Burn Game, powered by Kahoot (Kahoot! [2025]. Kahoot! Available on https://kahoot.com). The Burn Game was developed as a pedagogical tool to assess student learning through a point-based application system. A total of 25 multiple-choice questions were constructed and administered in both Pre-Test and Post-Test formats. Following each response, students received immediate feedback regarding the correctness of their selected alternative, thereby reinforcing conceptual understanding. The gamification framework was designed to foster team-based competition, with the content of the questions derived from the lecture content, including fundamental principles related to burn injury accidents and the identification of key factors that could guide the appropriate referral of burn victims to healthcare services, fostering teamwork and active participation. This step lasted approximately 30-minutes and encouraged collaboration and deeper understanding among peers.

Following these two steps, with an interval of 20-minutes, the same 21-question online form related to BIs was re-administered to the students, this time serving as the Post-Test. Completing this final step took an additional 20-minutes, allowing us to evaluate the effectiveness of the activity.

### Endpoints

The primary endpoint for the authors was the Online Form Score. Additionally, a subgroup comparison was conducted to evaluate the influence of educational programs on burn-related knowledge.

### Statistical analysis

Statistical analyses were performed using RStudio (RStudio Team, 2020). Given the design of this self-controlled study, an independent *t*-test was conducted to compare the mean scores between the Pre- and Post-Tests. The results are presented both as Mean Differences (MD) with 95% Confidence Intervals (95% CI) and as mean ± Standard Deviation (SD). Both violin and box plots were generated to provide a clearer visualization of score distributions across Pre- and Post-tests, as well as between HS and VS students. They were also made using RStudio.

### Sample size calculation

A preliminary analysis was conducted with 20 participants to support sample size estimation. Based on the data obtained, a final required sample size of 120 participants was calculated using STATA v18 (StataCorp, 2023. Stata Statistical Software: Release 18. College Station, TX: StataCorp LLC).

## Results

A total of four schools were included in the present analysis, each represented by a class selected by the school principal. Among these, one offered a VS course, whereas the remaining three provided HS courses. Specifically, the analysis encompassed two first-year and one second-year high school classes. Although all schools were public institutions, they were distributed across distinct regions of São Paulo. This distribution aimed to minimize potential biases arising from local disparities, which are particularly relevant given the city’s vast size and pronounced social inequalities.

Initially, 164 students were recruited for the study. However, 30 were excluded due to the absence of a properly completed ICF. Since this was a self-controlled study, each participant contributed to both the Pre- and Post-Test groups. Consequently, the authors analyzed a total of 268 form responses for our primary endpoint, despite the study involving only 134 participants.

### Population

Out of the 134 participants included in this study, 73 (54.5%) were enrolled in VS courses, and 98 (73.1%) were female. The mean age of the participants was 18.6 ± 4.9 years, with the high standard deviation largely attributable to the VS cohort. For a detailed overview of the demographic data of the 134 students included in our statistical analysis, please refer to Table 1.

**Table 1:** Study population baseline characteristics.

	Vocational School (n = 73)	High School (n = 61)	Total (n = 134)
**Male, n/N (%)**	20 (27.4%)	16 (26.2%)	36 (26.9%)
**Female, n/N (%)**	53 (72.6%)	45 (73.8%)	98 (73.1%)
**Age (yr, SD)**	20.2 ± 6.1	16.6 ± 1.1	18.6 ± 4.9

### Online form score

**Fig. 1:**
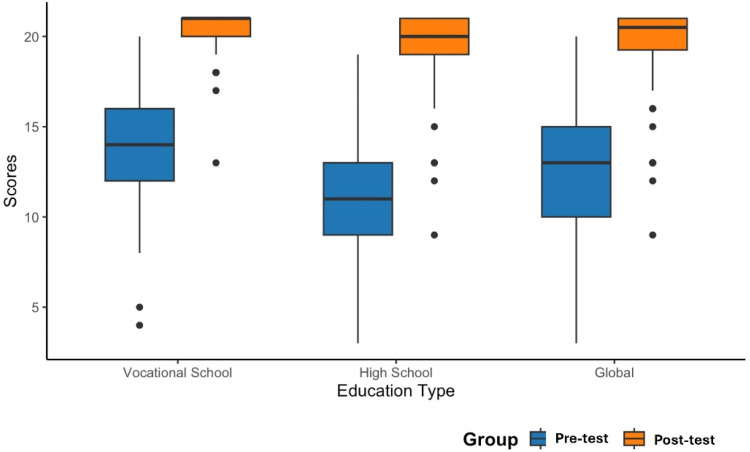
Box plot depicting pre-test and post-test scores, both overall and stratified by high school and vocational school students.

The primary endpoint of interest was the difference between Pre‑Test and Post‑Test scores. Post‑Test performance improved significantly among participants, with 132 out of 134 students achieving higher scores. Globally, the authors observed a marked increase in performance, with an MD of 7.16-points (95% CI: 6.47 to 7.86; p < 0.001). This substantial improvement was also evident in subgroup analyses: students enrolled in HS courses showed the most pronounced gains (MD = 8.46; 95% CI: 7.41 to 9.51; p < 0.001), while those in the VS cohort also demonstrated a consistent and meaningful increase (MD = 6.08; 95% CI: 5.33 to 6.83; p < 0.001). These results are presented numerically in Table 2 and visually depicted in Fig. 1 through a box-plot diagram.

**Table 2:** Scores of pre- and post-test of the study groups.

	Vocational School (n = 73)	High School (n = 61)	Total (n = 134)
**Pre-Test Score**^a^	14.2 ± 3.0	10.8 ± 3.4	12.7 ± 3.6
**Post-Test Score**^a^	20.3 ± 1.3	19.3 ± 2.5	19.8 ± 1.9
**MD (95% CI)**	6.08 (5.33 – 6.83)	8.46 (7.41 – 9.51)	7.16 (6.47 – 7.86)

a Mean ± SD.

### Educational program comparison

The analyses revealed that, prior to the intervention, the mean scores of the VS group were significantly higher than those of the HS group (MD = 3.34; 95% CI: 2.25 to 4.43; p < 0.001).

Also, the HS group learned a lot more after the intervention than the VS group (MD = 2.38; 95% CI: 1.21 to 3.55; p < 0.001). This suggests that the type of educational program may affect both students’ baseline knowledge and their responsiveness to the intervention.

**Fig. 2:**
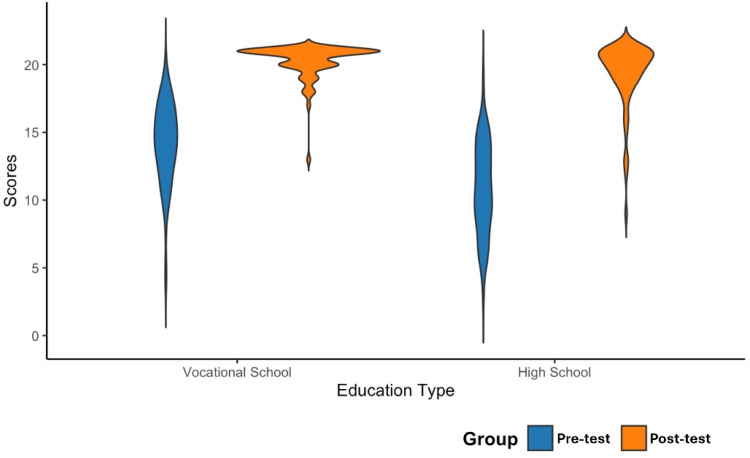
Violin plot showing the distribution of pre- and post-test scores of study groups.

A violin plot was created to further illustrate these findings (Fig. 2). As shown, Pre-Test scores for HS students were relatively evenly distributed along the y-axis but rarely reached the highest grades. In contrast, Pre-Test scores for VS students were concentrated around 15, with some reaching the top clusters. Post-Test scores for VS students shifted to the highest clusters, showing a strong consolidation of knowledge. Similarly, HS students displayed numerous scores in the second-highest cluster, with a notable presence in the top cluster as well. In both groups, the Post-Test shows a clear shift towards higher scores, highlighting the effectiveness of the educational activity.

## Discussion

Serious games are being used more and more in learning models because they are proven to improve motivation, engagement, interest, and learning outcomes, especially among Generation Z.^10^, ^11^, ^12^ For this reason, the authors suggested using a game-based approach to teach Brazilian HS and VS students about burns. This segment of the population has no prior knowledge of burn injuries, and Generation Z, easily reached through schools, represents a key target for burn prevention initiatives. In addition to being at risk themselves, adolescents and young adults play a critical role in the care and protection of children, who account for 48% of all burn injuries.^7^

The literature review revealed a scarcity of studies investigating the use of serious games for burn prevention education. One example is the study by Burgess et al. (2018),^9^ which conducted a randomized trial using the “Cool Runnings” mobile app, designed to prevent scald burns in infants aged 5- to 12-months. The app targeted mothers and incorporated serious game elements in the intervention group, which was compared to a standard version without gamification. The gamified group demonstrated greater knowledge acquisition; however, the study experienced a high dropout rate (approximately 51%), which may have led to an overestimation of the app’s effectiveness.^9^

Designed to be both engaging and effective, the intervention consisted of a structured 50-minute in-person session. It began with a 20-minute lecture aimed at reinforcing students’ foundational knowledge by addressing all the layperson-oriented topics assessed in the pre-intervention test. The in-person format contributed to minimizing participant dropout, ensuring greater adherence throughout the activity. The Burn Game represents the first iteration of a research line focused on gamification.

The study population consisted of 134 students enrolled in either VS or HS programs at Brazilian public institutions. Among them, 73 participants (54.5%) were from the VS, and 61 (45.5%) were from three HSs, reflecting a reasonably balanced distribution. A total of 98 participants (73.1%) were female. This disproportion may reflect a baseline selection bias, as previous studies suggest that female students tend to be more engaged in digital environments.^13^ This factor may have contributed to the higher participation of female students in the present sample. However, it does not necessarily indicate a differential response to the intervention itself, and it may instead represent a limitation concerning the generalizability of these findings. Lastly, the mean age of participants was 18.6±4.9 years, with the high standard deviation primarily driven by the VS group. In Brazil, vocational programs often include both adolescents who are concurrently enrolled in HS and young adults seeking technical training, contributing to the broader age range.

The group selected public schools based on population representativeness. In Brazil, low-income families commonly enroll their children in public schools, as the average income often renders private education financially inaccessible. Unfortunately, public schools in the country frequently face limitations in infrastructure and access to high-quality educational resources.^14^ Implementing this game-based model in public schools was not only a strategy to reach vulnerable populations in a context with reduced risk of dropout and selection bias, but also an effort to generate meaningful social impact.

The scores from the online form underscore the effectiveness of the gamified educational intervention in enhancing students’ knowledge about BIs. Globally, the intervention led to a significant increase in scores (MD=7.16; 95% CI: 6.47 to 7.86; p<0.001), reflecting the combined impact of the lecture and the Burn Game in reinforcing learning. This result aligns with expectations, as gamified educational models have consistently demonstrated effectiveness across various fields, particularly when applied to adolescents and young adults.^10^, ^11^, ^12^ This age group’s familiarity with digital tools is undeniable and provides a valuable opportunity for engagement, as exemplified by the Burn Game.

The authors also analyzed HS and VS separately, and this analysis revealed notable differences between HS and VS students. The HS group exhibited a greater relative improvement (MD=8.46; 95% CI: 7.41 to 9.51; p < 0.001) compared to the VS group (MD = 6.08; 95% CI: 5.33 to 6.83; p < 0.001), likely due to the lower baseline scores observed among HS students. However, VS students achieved higher absolute post-intervention scores, which may be attributed to their technical educational background. Notably, the VS program analyzed in this study focused on chemistry, a subject closely related to the content of the intervention, which likely contributed to their higher baseline knowledge. This specific focus may have amplified the differences in baseline scores between the two groups, emphasizing the influence of educational context on pre-intervention knowledge.

These findings suggest that the intervention is broadly effective, but that low baseline knowledge levels significantly influence the relative gains observed. The greater improvement among HS students highlights the potential of gamified models to address knowledge gaps effectively. Meanwhile, the substantial yet smaller improvement in the VS group reflects the intervention’s ability to refine and build upon existing knowledge. These trends are visually represented in the violin plot (Fig. 2), which illustrates the upward shift in scores for both groups post-intervention, while also highlighting the role of the educational program in shaping pre- and post-intervention outcomes.

Despite the promising results, the present study has some limitations that must be acknowledged. First, it was not possible to determine the extent to which the observed improvement in test scores was attributable to the gamified model versus the lecture component. It is reasonable to assume that the improvement resulted from the combined effect of both strategies, with the lecture facilitating content exposure and the game reinforcing retention. However, testing this hypothesis would require longer follow-up periods, which was unfeasible given the structural constraints of the Brazilian public education system.

To overcome this limitation, the group is currently collaborating with partner institutions to develop mobile app-based versions of the game. These would allow for extended engagement and enable follow-up assessments. Furthermore, reliability must be formally assessed; in the next phase of the study, the authors plan to re-administer the questionnaires after a seven-day interval. Finally, the authors aim to design a randomized clinical trial that will include a control group (lecture only) as a proof of concept to more rigorously evaluate the influence of the gamified component on the effectiveness of the intervention. Additionally, the authors observed a dropout rate of approximately 18%, primarily due to incomplete informed consent submissions.

## Conclusion

Overall, the present findings suggest that the gamified approach is effective in educating young students about BIs. However, its effectiveness in preventing such injuries could not be assessed, as evaluating knowledge retention and practical application would require a longer follow-up period. Future projects within this line of research aim to incorporate extended follow-up through serious games developed in partnership with other institutions.

## Author's contribution

Felipe Giraldo Alvarez Gonçalves: data analysis, data curation, writing; Vinicius Oberdan Souza Reis: data analysis; Vinicius Freire Costa Alves: data analysis; José Maria Soares Junior: review; Sérgio Henrique Bastos Damous: Review; Patricia Zen Tempski: writing and review; Edivaldo Massazo Utiyama: Review; Rolf Gemperli: supervision; Cristina Pires Camargo: conceptualization, supervision.

## Funding

This study had no funding.

## Data availability

The datasets generated and/or analyzed during the current study are available from the corresponding author upon reasonable request.

## Conflicts of interest

The authors have no financial or non-financial interests to disclose.
